# Chronic Comorbidities Contribute to the Burden and Costs of Persistent Asthma

**DOI:** 10.1155/2015/819194

**Published:** 2015-12-13

**Authors:** Paula Kauppi, Miika Linna, Juha Jantunen, Jaana E. Martikainen, Tari Haahtela, Anna Pelkonen, Mika Mäkelä

**Affiliations:** ^1^University of Helsinki, Helsinki University Central Hospital, Respiratory Diseases and Allergology, Inflammation Center, Skin and Allergy Hospital, P.O. Box 160, 00029 Helsinki, Finland; ^2^Department of Industrial Engineering and Management, Aalto University, P.O. Box 15500, 00076 Aalto, Finland; ^3^South Karelia Allergy and Environment Institute, Lääkäritie 15, 55330 Tiuruniemi, Finland; ^4^Social Insurance Institution, Research Department, P.O. Box 450, 00101 Helsinki, Finland; ^5^University of Helsinki, Helsinki University Central Hospital, Pediatric Diseases and Allergology, Inflammation Center, Skin and Allergy Hospital, P.O. Box 160, 00029 Helsinki, Finland

## Abstract

*Background*. We aimed to study the prevalence of chronic comorbidities in asthma patients and the costs of health care use associated with asthma with comorbidities.* Material and Methods*. We analysed the prevalence of the four most common chronic diseases in asthma patients in 2008–2014 in Finland. Prevalence of coronary artery disease, diabetes and dyslipidaemia, hypertension, epilepsy, inflammatory bowel disease, rheumatic diseases, and severe psychiatric disease was studied by register of the Social Insurance Institution of Finland. The costs of health care services were collected from the registries maintained by the National Institute for Health and Welfare (THL).* Results*. Prevalence of asthma was 4.6% in 2014. Diabetes was among the four most common comorbidities in all the age groups. The other common comorbidities were hypertension (≥46 years; 12.9–37.6%), severe psychiatric disorders (age groups of 16–59 years; 1.4–3.5%), and ischaemic heart disease (≥60 years; 10–25%). In patients with both asthma and diabetes, the costs of hospitalization were approximately 169% compared with patients with asthma alone.* Conclusions*. Prevalence of asthma increases by tenfold when aging. The comorbidity diversity and rate are age-dependent. Prevalence of diabetes as comorbidity in asthma has increased. Costs of hospitalizations in asthma approximately double with chronic comorbidities.

## 1. Introduction

Early diagnosis of asthma and effective asthma medication together with well-organized control options, patient education, and self-management plans have led to major decrease in asthma hospitalizations [1, 2]. Nonadherence to antiasthmatic medication, long-term smoking, and poor lung function are risk factors for emergency department visits [3, 4]. We have earlier demonstrated that asthma hospital days are highest in 0–5-year olds and in older than 75-year olds [5].

Usually comorbidity in asthma has been referred as obesity, depression, or anxiety, reflux disease, or different atopic disorders such as allergic rhinoconjunctivitis or atopic eczema. Mostly asthma has been presented as a part of atopic march or atopic constitution. Similarly, generalized systemic inflammation has been represented as a cause for chronic obstructive pulmonary disease and associated comorbidities such as coronary artery disease and osteoporosis. If searching for explanations for increased hospitalization rate in elderly asthma patients, atopic disorders, obesity, or reflux disease are unlikely to be causative factors and thus other systemic diseases or associated comorbidities should be studied.

In here, we searched for explanations for marked increase in asthma hospitalizations in the elderly by studying the prevalence and variety of comorbidities of asthma patients and health care costs associated with health care use. Comorbidities were assessed as chronic diseases entitling patients to higher than regular reimbursements for their drug costs by the Finnish national health insurance.

## 2. Material and Methods

We explored what are the other chronic, comorbid diseases in the Finnish asthma patients during a time period from 2008 to 2014. For that we used the nationwide Special Reimbursement Register maintained by the Social Insurance Institution (SII). In Finland, patients with defined chronic diseases (e.g., asthma, diabetes, rheumatoid arthritis, coronary artery disease, and hypertension) are entitled to higher than regular reimbursement (special reimbursement) for their drug costs. To be eligible for the higher reimbursement, the patient must obtain a doctor's certificate to confirm the diagnosis and regular need for medication for persistent disease.

There are strict criteria for the special reimbursement of asthma medication. The disease is required to be diagnosed according to the international guidelines and variable airflow limitation objectively shown according to the following criteria: (1) forced expiratory volume in 1 second (FEV1) improves at least 12% (200 mL) after inhaling *β*2-agonist or peak expiratory flow (PEF) improves at least 15% (60 L/min), (2) PEF follow-up indicates diurnal variation of at least 20% or at least 20% improvement in PEF level during asthma treatment, or (3) bronchial responsiveness to histamine or methacholine is moderately or severely increased [3]. Asthma patients were identified by the ICD10 classification codes for J45 and J46.

Comorbidities to asthma were included by the Social Insurance Institution special reimbursement for the according comorbidity. The four most common comorbidities to asthma were analysed. Comorbidity groups included diagnoses for diabetes (E10–E14, E89.1), rheumatoid arthritis, and other connective tissue diseases (A04.6, A39.8, A50.5, D76.0, D76.3, H20.1, H30, I33.0, J84, K50.9, K51.9, K73.2, K74.3, K83.0, L40.5, M02, M05, M06, M08, M13.9, M30–M35, M45, M46.1, M46.9, M94.1, N03, and Q44.2), coronary artery disease (I20–I22, I24.0, and I25), and hypertension (I10–I13, I15, and I27.0) (Fimea). In addition, we searched for severe psychiatric disorders (A52.1, A69.2, A81.0, B22.0, B56.9, B57.2, E01.8, E03.9, E52, E53.8, E75.6, E83.0, E83.5, F01, F03, F20–F25, F28, F29, F30.2, F31, F32.3, F33.3, F84, G10, G20, G30.0, G30.1, G30.8, G30.9, G31.0, G35, G40.9, M30.0, and M32.8), dyslipidemia associated with coronary artery disease (I20–I22, I24.0, and I25), colitis ulcerosa and morbus Crohn (K50, K51), and epilepsy (C71, G40, and G41).

Data for use of health care services were collected from the National registries maintained by the National Institute for Health and Welfare (THL). These registers included the hospital benchmarking database [6] and the national discharge registry (HILMO, [7]). Secondary care data included the use of hospital outpatient care (all types of hospital visits) and hospital inpatient admissions (DRGs). The DRG costings for hospitalizations and outpatient visits were based on individual-level cost accounting data from several hospitals and the same cost weights are used in the standard price list reported by THL in the national level.

Standard population sizes in different age groups were obtained from Statistics Finland [8].

Proportions (percentages) of asthma patients were calculated from the total population and from the number of people in the relevant age group. The number of asthma patients with the comorbidity in question was adjusted for the standard population size (100 000) according to the age group. The four most common comorbidities to asthma were analysed. The distribution of chronic diseases was calculated by chi square test and using the Bonferroni correction for multiple testing. Pearson correlation was used to analyse correlation of asthma and diabetes. *P* value < 0.05 was considered statistically significant.

## 3. Results

**Figure 1:**
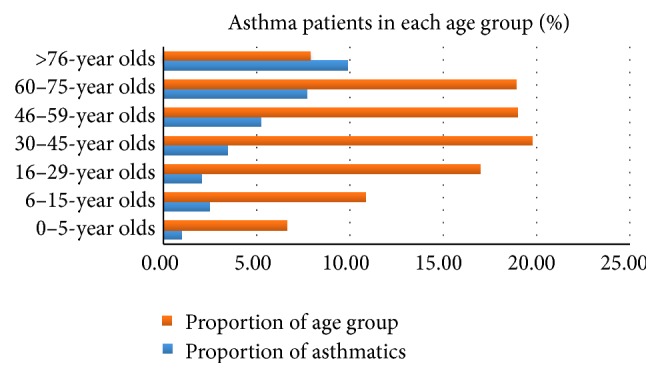
Proportion of asthmatics in different age groups and proportion of the age group of total population in 2014.

In 2014 the number of asthmatics in the special reimbursement register was 251 540 (4.6% of the total Finnish population). The absolute and relative number of asthmatics increased with age as did the number of those with various comorbidities (Figure 1, Table 1). In the oldest age group approximately half of the asthma patients had another chronic disease. Percentage of patients with persistent asthma increased from 0–5-year olds (0.97%, 969 incident cases per 100 000 individuals of the whole population of the studied age group) to 46–59-year olds (5.2%, 5236/100 000), to 60–75-year olds (7.7%, 7705/100 000) and to over 75-year olds (9.9%, 9897/100 000).

**Figure 2:**
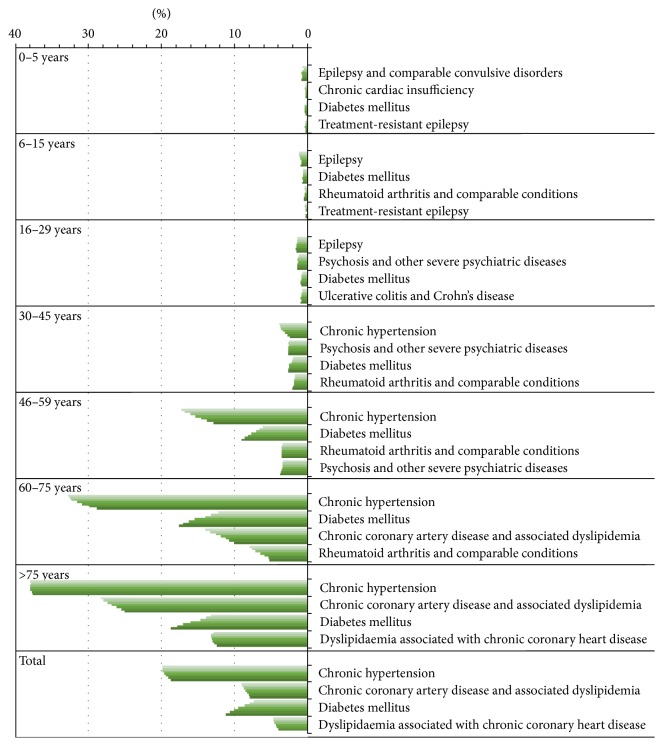
Four most common comorbidities among patients with persistent asthma in different age groups according to Social Insurance Institution register data from 2008 to 2014 (from top and light grey to bottom and dark green of a bar).

The most common chronic comorbidity in the three oldest age groups was (46 years or older)* hypertension* with prevalence increasing from 12.9 to 37.6% (676/100 000; 2228/100 000 and 3723/100 000 individuals) according to group (Table 1, Figure 2).* Diabetes* was among the four most common comorbidities in all the age groups and its prevalence was 17.5–18.8% in the two oldest age groups of asthma patients (≥60 years) (1353/100 000 and 1856/100 000 individuals). The other common comorbidities were* severe psychiatric disorders* with the prevalence varying from 1.4 to 3.5% (29/100 000 individuals, 91/100 000 and 183/100 000) (three age groups of asthma patients 16–59 years) and* ischemic heart disease* with the prevalence varying from 10.0 to 25.0% (774/100 000 and 2474/100 000) (two age groups of asthma patients of ≥60 years (Table 1, Figure 2)).

Proportion of asthma patients with at least one comorbidity increased considerably with age. In the oldest age group (75 years or older), 37.6% had hypertension (3223/100 000), 25.0% ischemic heart disease (2474/100 000), 18.8% diabetes (1856/100 000), and 12.4% dyslipidaemia (1226/100 000) (Table 1, Figure 2). In asthmatics, the distribution of the four most common other reimbursed chronic diseases was significantly different in all age groups (*P* < 0.00001) compared with the total population, also after Bonferroni correction for multiple testing.

**Figure 3:**
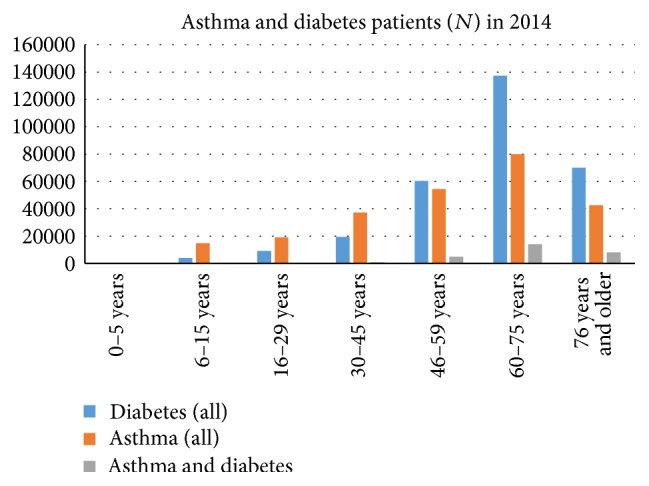
Number and distribution of asthma and diabetes patients according to age.

Asthma patients with diabetes had increased from 2008 to 2014 which is a trend found also in general population. Diabetes increased from 12.3% to 17.6% (*P* < 0.001) in the 60–75-year-old asthmatics and from 13.3% to 18.8% in those over 75 years (*P* < 0.001) (Figure 2). Prevalence of diabetes (both type I and type II) was 5.5% in the total population and 11.2% among all the asthma patients, thus showing a twofold prevalence among asthmatics (Figure 3, Table 2). Prevalence of asthma correlated with diabetes (*r* = 0.936, *P* = 0.002).

In contrast, hypertension decreased in the 46–59-year-old asthmatics from 17.3% to 12.9% during the same time period (*P* < 0.001). In spite of the increased prevalence of ischemic heart disease in older age groups the prevalence of reimbursed chronic coronary artery disease decreased in the age group of 60–75-year-old asthmatics from 14.5% to 10.0% (*P* < 0.001).

During the observed period, the costs of hospitalizations caused by asthma were approximately 1217 €/year/patient. In patients with both asthma and diabetes together, the costs were approximately 69% higher compared to those with asthma only. In patients with asthma and ischaemic heart disease the costs of hospitalizations were 191% higher compared to asthma alone and in asthma and heart insufficiency the costs were 259% higher than in those with asthma alone.

## 4. Discussion

Prevalence of asthma increases from 0.97% to 9.9% when aging. Comorbidities to asthma were different in the youngest and the oldest age groups except for diabetes. In addition, the prevalence of comorbidities increases in older populations accordingly. In the 0–5-year-old asthma patients, 0.20% had epilepsy or other comparable convulsive disorders. In our study, 28.9% of the 60–75-year-old asthma patients and 37.6% of at least 75-year-old asthma patients had chronic hypertension. The most often found comorbidities differed according to the age groups, but hypertension, chronic coronary heart disease, diabetes, and severe psychiatric disorders were the most often associated chronic diseases in patients with asthma. Percentage of asthmatics with diabetes had increased and was 11.2% in the total asthma population whereas prevalence of diabetes was 5.5% in the total population.

These nationwide statistics on medication compensation are in accordance with the information obtained from questionnaire studies showing that asthma is the one of the most prevalent chronic diseases of all ages and that chronic comorbidities of asthma patients follow the distribution of chronic diseases in overall population. Epilepsy or other comparable convulsive disorders were found in the youngest age groups of asthma patients; severe psychiatric disorders, diabetes, and rheumatoid arthritis were in the middle age groups whereas chronic hypertension, chronic coronary heart disease, and diabetes were among the most common chronic diseases in the oldest age groups of asthma patients. Having diabetes as comorbidity to asthma increased the hospitalization costs by 69%.

In France, 40.2% of the patients with severe asthma had at least one chronic comorbidity [9]. Every other US adult asthma patients have been reported to have at least one other chronic condition [10]. In our study, 37.6% of 75-year-old or older asthma patients had chronic hypertension which was the most common single chronic disease in asthma patients.

In a Norwegian study, 8–19-year-old male asthmatics had 4.6 times increased odds ratio for gastrooesophageal reflux disease (GERD), 4.2 for allergy, and 1.8 for epilepsy. In female asthmatics of the same age, odds ratio was increased by 4.8 for having allergy, by 4.0 for having GERD, and by 2.0 for having epilepsy [11]. Epilepsy was the most common chronic disease in our study in age groups from 0 to 29 years. In a US child study population aged 0–17 years odds for having epilepsy were 2.3 in those with lifetime prevalence of asthma and 2.0 in those with one-year prevalence of asthma [12]. However, the possible mechanisms for this association are unknown. In older age groups other diseases than epilepsy were more prominent. Further, the prevalence of allergy and gastrooesophageal disease could not be estimated in our study since neither entitles patients to special reimbursements for drugs costs. However, both allergy and GERD are widely accepted and known disorders in asthmatics and lately GERD has been associated with neutrophilic asthma [9, 13–15]. In the same Norwegian study it was shown that odds were increased by 1.6–2.1 for purchasing antibacterial and antiviral drugs in asthma patients. Our study was limited only to data on chronic diseases which do not include short courses of antibacterial and antiviral drugs.

Further, in a Canadian population-based study on asthma and health care service use, comorbidities were associated with 5.5–7.6% of ambulatory claims, emergency department visits, and hospitalizations [16]. In a later paper of Gershon et al., comorbidity to asthma was studied by physician claims. Also in their study spectrum of other diseases to asthma varied in different age groups. Other respiratory disorders than asthma (acute bronchitis, pneumonia) were common in all ages [17]. Psychiatric disorders and metabolic and immunity disorders were often found causes for physician claims in the youngest age group. Our results are in accordance since epilepsy, diabetes, and rheumatic diseases were the most common found disorders in our study in 0–15-year-old asthmatics. Psychiatric disorder and infectious diseases were the most common disorders in the age groups of 18–44- and 45–64-year-old asthmatics for physician claims in the Canadian study [18]. In our study, psychiatric disorders were among the four most common chronic comorbidities in 16–59-year-old asthmatics.

If focusing on older age groups, we found that chronic hypertension, diabetes, coronary disease, and hyperlipidaemia are the most common comorbidities. In a study of severe asthma patients of mean age of 56 years 11.9% of the asthmatic had diabetes, 16.5% osteoporosis, and 21.1% hypertension [9]. This is also in accordance with our results with diabetes being the second or third most common comorbidity in those of 60 years or older (17.6–18.8%) and hypertension being the most common chronic comorbidity, accordingly (prevalence 28.9–37.6%).

Obesity is associated with asthma both as a risk factor for severe asthma and as a risk factor for development of asthma [19, 20]. Further, whether asthma could lead to morbid obesity has also been suggested, for example, by decreasing physical activity. Lately, a common pathogenetic cause for both asthma and obesity has been introduced [21, 22]. Chitinase 3-like 1 (CHI3L1) deficient mice have been reported to develop less visceral obesity. Likewise, overexpression of CHI3L1 induced obesity and increased allergic airway inflammation in association with high-fat diet. Increasing obesity at the population level is also the main cause for increasing prevalence of type II diabetes. In this study, we could not distinguish between type I and type II diabetes or characterize severity of asthma. A link between asthma and diabetes has been introduced not only in predisposition but also in treatment of these diseases. In a recent study, insulin was shown to suppress inflammatory pathway in obese individuals with type II diabetes [23]. Insulin decreased mRNA expression of IL-4, ADAM-33, and LTBR (lymphotoxin beta receptor), which are all potentially involved in pathogenesis of asthma.

Although both asthma and rheumatoid arthritis are inflammatory diseases they are seldom described to be associated probably because asthma is usually associated with Th2-type inflammation and rheumatoid arthritis is considered to be originated of Th1-type inflammation. In a Taiwanese study with 27 602 patients with rheumatoid arthritis, asthma incidence was reported to be twice (4.56 per 1000 person-years) compared to the general population (2.22 per 1000 person-years) [24]. We studied prevalence and not incidence, but rheumatoid arthritis was among the four most common chronic comorbidities in asthma patients in 6–15-year olds and 30–75-year olds. In 16–29-year old asthmatics, other inflammatory diseases such as ulcerative colitis and Crohn's disease were the third most common comorbidities.

Asthma has been associated with anxiety and depression in many studies [25, 26]. In a recent meta-analysis of allergy related cytokines in depression, IL1, IL4, IL6, and TNF-*α*, were found increased in depression compared to nondepressive study subjects and were considered to be a possible inflammatory link between the two disorders [27]. A possible common susceptibility to both asthma and anxiety or depression either in the form of inflammatory mechanisms or genetic factors is supported by an epidemiologic study by Goodwin et al. [25]. They found increased odds for prevalence of concurrent or persistent anxiety and depression in patients with respiratory disease. In our study, 3.5% of the 46–59-year-old asthma patients suffered from psychosis or other severe psychiatric diseases and had received reimbursement for both disorders.

Morbidity increases together with age and, accordingly, the rate of comorbidities in asthma patients is age-dependent. The World Health Organization (WHO) has estimated that noncommunicable diseases account for 63% of all deaths [28]. In our study, chronic hypertension, diabetes, coronary disease, and hyperlipidaemia were the most common comorbidities to asthma following age-dependent disease distribution of a normal population. Both asthma and diabetes affect individuals of all age groups, with asthma being more common in children and adolescents and diabetes in the elderly (Figure 3). Although asthma is among the four most common noncommunicable diseases (cardiovascular diseases, chronic respiratory diseases, cancers, and diabetes) it is noteworthy that malignancies were not found in our study for comorbidities in asthma not even in the oldest age groups.

Medication costs of asthma have increased while hospitalizations and physician visits have decreased [1, 18]. However, it is known that hospitalizations accumulate in the youngest and in the oldest age groups of asthma patients. Further, less is known of the costs' increase associated with comorbidity. Here, we found an increase from 169% to 359% in asthma hospitalizations associated with chronic comorbidity.

## 5. Conclusions

Prevalence of asthma increases by tenfold when aging. The most common other reimbursed chronic conditions in addition to asthma in the youngest age groups were epilepsy or other comparable convulsive disorders. In the middle aged asthmatics, severe psychiatric disorders, diabetes, and rheumatoid arthritis predominated. Hypertension, coronary heart disease, and diabetes were among the most common chronic diseases in the oldest age groups of asthma patients. Hospitalizations in persistent asthma increase considerably in the age group over 75 years compared to other age groups and it is possible that chronic cardiovascular comorbidity has a major role in the hospitalizations associated with asthma in the oldest age group. Chronic diseases as comorbidity increase significantly the costs of hospitalizations in asthma patients.

## Figures and Tables

**Table 1:** The four most common comorbidities in asthma patients in different age groups according to Special Reimbursement Register. Total numbers of asthmatic patients, asthmatic patients with the disease, and all the patients with the diseases in year 2014.

Age group	Disease	Number of patients with chronic disease and asthma	Number of patients with chronic disease	Number of patients with asthma and special reimbursement for medication	Total population
0–5-year olds	Epilepsy and comparable convulsive disorders	28	713	3 508	362 128
	Chronic cardiac insufficiency	11	158		
	Diabetes mellitus	10	450		
	Treatment-resistant epilepsy	9	245		

6–15-year olds	Epilepsy	135	2 307	14 746	594 087
	Diabetes mellitus	101	3 923		
	Rheumatoid arthritis and comparable conditions	76	1 714		
	Treatment-resistant epilepsy	43	794		

16–29-year olds	Epilepsy	285	8 769	19 099	929 208
	Psychosis and other severe psychiatric diseases	268	8 819		
	Ulcerative colitis and Crohn's disease	176	5 183		
	Diabetes mellitus	161	9 132		

30–45-year olds	Psychosis and other severe psychiatric diseases	982	20 723	37 292	1 082 178
	Diabetes mellitus	976	19 326		
	Chronic hypertension	919	12 905		
	Rheumatoid arthritis and comparable conditions	771	14 483		

46–59-year olds	Chronic hypertension	7 031	83 251	54 439	1 039 779
	Diabetes mellitus	4 905	60 454		
	Rheumatoid arthritis and comparable conditions	2 046	24 895		
	Psychosis and other severe psychiatric diseases	1 901	26 901		

60–75-year olds	Chronic hypertension	23 081	224 435	79 825	1 036 018
	Diabetes mellitus	14 013	137 319		
	Chronic coronary artery disease and associated dyslipidemia	8 016	76 456		
	Rheumatoid arthritis and comparable conditions	4 146	38 588		

76-year olds	Chronic hypertension	16 037	145 447	42 634	430 757
	Chronic coronary artery disease and associated dyslipidemia	10 657	84 940		
	Diabetes mellitus	7 995	70 104		
	Dyslipidaemia associated with chronic coronary heart disease	5 282	42 642		

**Table 2:** Distribution of asthma and diabetes patients in the total population in 2014.

Age	Diabetes (all)	% (of the total population)	Asthma (all)	% (of the total population)	Asthma and diabetes	% (of total population)	Total population
0–5 years	450	0.12	358	0.098	10	0.0028	362128
6–15 years	3923	0.66	14746	2.5	101	0.017	594087
16–29 years	9132	0.98	19099	2.1	161	0.017	929208
30–45 years	19326	1.8	37292	3.4	976	0.09	1082178
46–59 years	60454	5.8	54439	5.2	4905	0.47	1039779
60–75 years	137319	13.3	79825	7.7	14013	1.4	1036018
76 years and older	70104	16.3	42634	9.9	7995	1.9	430757
